# Effect of *Nitya-Abhyaṅga* (self-massage) combined with stretching on hamstring extensibility, muscle stiffness, and stretch tolerance in college students: a randomized controlled trial

**DOI:** 10.3389/fspor.2026.1800782

**Published:** 2026-06-01

**Authors:** Tony Jacob John, Anusree Dileep, Vandana Rani Madhavan, Meera Sudha, Delvin T. Robin

**Affiliations:** Department of Swasthavritta (Social and Preventive Medicine), Amrita School of Ayurveda, Amritapuri, Amrita Vishwa Vidyapeetham, Kollam, India

**Keywords:** *Abhyaṅga*, ayurveda, college students, hamstring flexibility, muscle stiffness, sedentary lifestyle, self-massage, stretching exercise

## Abstract

**Introduction:**

Prolonged sitting is increasingly prevalent among young adults and is associated with reduced hamstring extensibility, increased stiffness, and heightened injury risk. Although stretching exercises can improve range of motion, their effects are often transient, with limited effect on muscle stiffness. *Nitya-Abhyaṅga* (daily self-massage) is a traditional *Ayurvedic* practice indicated for stiffness and restricted movements. However, evidence regarding its effect on hamstring flexibility remains limited. This study evaluated the combined effect of *Nitya-Abhyaṅga* and stretching exercises on hamstring extensibility, stiffness, and stretch tolerance in college students.

**Method:**

In this randomized controlled trial (CTRI/2024/07/070623), 42 male college students aged 18−25 years with reduced hamstring extensibility (hand–toe distance >0 cm) were randomly allocated, after obtaining informed consent, to either Group A (*Nitya-Abhyaṅga* with stretching) or Group B (stretching alone) for a 4-week intervention period. Outcomes included the hand–toe test, knee extension test, hip flexion angle, muscle stiffness assessed using a digital algometer, and stretch tolerance measured using a visual analog scale, measured at baseline and post-intervention, with compliance monitored through participant diaries.

**Result:**

Both groups demonstrated significant improvements over time. Two-way repeated-measures ANOVA revealed significant group ×  time interactions across all outcome measures (*p* ≤ 0.001), indicating significant improvement in the *Abhyaṅga* with stretching group compared with stretching alone, with moderate to large effect sizes.

**Conclusion:**

*Nitya-Abhyaṅga* combined with stretching was more effective than stretching alone in improving hamstring extensibility, reducing algometer-derived stiffness, and enhancing stretch tolerance. This combined approach may be recommended as a simple, cost-effective preventive practice to mitigate the adverse effects of prolonged sitting and to maintain musculoskeletal health.

**Clinical Trial Registration**: identifier CTRI/2024/07/070623, https://ctri.nic.in/Clinicaltrials/pmaindet2.php?EncHid=MTExODU3&Enc=&userName=

## 1 Introduction

The hamstring muscle complex consists of a group of three individual muscles—the semitendinosus, semimembranosus, and biceps femoris—located in the posterior compartment of the thigh (1). Together, they are involved in activities ranging from standing and walking to sprinting and jumping (2). Functional activities of daily life require appropriate hamstring stiffness and extensibility; deficits may cause lower back injuries (3) and changes in sitting posture due to postural instability (4).

Sedentary behavior has become increasingly prevalent among young adults, with approximately 31% of individuals over 15 years of age engaging in sedentary lifestyles characterized by prolonged sitting, digital dependency, and reduced physical activity (5). Prolonged sitting, commonly defined as sitting for ≥6 h/day, has been linked to muscle stiffness and reduced hamstring extensibility (6). Prevalence studies indicate that reduced hamstring extensibility may be present in up to 73% of college students (7), with males demonstrating a 62% higher risk of hamstring strain and related injuries compared with females (8). These observations suggest that sedentary behavior is a significant risk factor for hamstring-related musculoskeletal disorders in young populations (9).

Prolonged physical inactivity contributes to increased connective tissue stiffness through structural and mechanical adaptations occurring at both subcellular and tissue levels (10). Reduced loading of skeletal muscle is associated with a decrease in sarcomere number and alterations in the elastic properties of muscle fibers (11). At the subcellular level, titin, a large elastic protein, adapts to loading and unloading patterns and demonstrates reduced extensibility with induced unloading in animal models (11, 12). At the tissue level, the extracellular matrix (ECM), which is rich in collagen, plays a key role in the mechanical properties of muscles (13). As ECM depends on loading patterns (14), reduced physical activity promotes increased connective tissue stiffness (15). Together, these changes result in reduced hamstring extensibility and impaired functional range of motion.

Furthermore, even in resting muscle, a small proportion of myosin filaments remain bound to actin filaments, forming weak yet “long-lasting” cross-bridges (16). These cross-bridges contribute to increased passive muscle tension (16, 17). Such mechanisms may further increase stiffness in the hamstring muscles of individuals with a history of prolonged sitting.

Stretching is one of the most widely practiced therapeutic and conditioning interventions to improve soft-tissue flexibility and functional range of motion (18). However, the benefits of stretching are primarily mechanical, acting through elongation of muscle and connective tissues, with limited effect on neuromuscular and viscoelastic properties that contribute to muscle stiffness (19). As a result, massage techniques have been explored as adjuncts to stretching to enhance tissue compliance and recovery. Hodgson et al. reported that a 10-min roller massage improved hamstring range of motion (20); however, electromyographic data suggested that massage produced only transient spinal motor neuron relaxation, lasting <1 min (21). This suggests that a single massage session may be insufficient to achieve sustainable improvement in flexibility. In contrast, Akazawa et al. demonstrated that daily self-massage for 3 min at the musculotendinous junction improved hamstring extensibility, although no corresponding reduction in muscle stiffness was observed, implying that regularity and duration are crucial in eliciting measurable benefits (19).

In Ayurveda, reduced muscle extensibility and increased stiffness correlate to the concepts of *Saṅkocha* (contracture) and *Stambha* (rigidity), which arise due to localized vitiation of *Vāta Doṣa* (22). *Abhyaṅga* (therapeutic oil massage), described as a part of *Dinacharya* (daily regimen to maintain health), is advocated as both a preventive and curative measure for *Vāta-*related disorders (23). *Ācārya Vāgbhaṭa* mentions that *Abhyaṅga* nourishes tissues (*Puṣṭi*), imparts strength (*Dārḍhya*) (23), and reduces stiffness (*Stambha*) and constriction (*Saṅkocha*) (24).

Despite growing interest in integrative approaches and extensive classical references, systematic evaluation of *Abhyaṅga* combined with stretching on hamstring extensibility, muscle stiffness, and stretch tolerance remains limited. Therefore, this trial aimed to investigate whether daily *Abhyaṅga* combined with stretching produces superior outcomes compared with stretching alone in college students with reduced hamstring flexibility.

## 2 Methodology

### 2.1 Ethics approval

This study protocol was approved by the Institutional Ethics Committee of Amrita School of Ayurveda (IEC.ASA.PGR/07/24/APPROVAL). The trial was prospectively registered with the Clinical Trial Registry of India (CTRI/2024/07/070623). All procedures were conducted in accordance with the Declaration of Helsinki, and written informed consent was secured from all participants prior to enrollment.

### 2.2 Study design and setting

**Figure 1:**
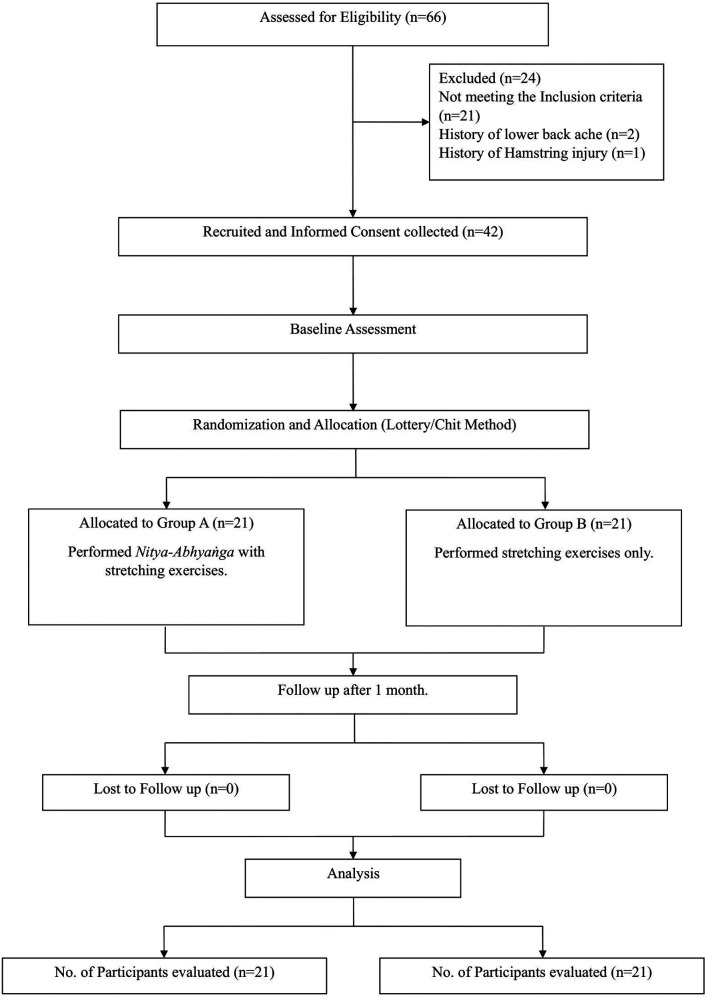
CONSORT guideline.

This study was an open-label, two-arm, parallel-group randomized controlled trial conducted at Amrita Vishwa Vidyapeetham, Amritapuri Campus, Kollam, Kerala, India, between March and October 2025. The intervention duration was 4 weeks (approximately 1 month). The study was conducted in accordance with the Consolidated Standards of Reporting Trials (CONSORT) guidelines (Figure 1).

### 2.3 Participants

Participants were male college students recruited from a single university through direct interaction. All participants were screened prior to enrollment. Recruitment was carried out between March and September 2025.

#### 2.3.1 Inclusion criteria

Participants were eligible for inclusion if they satisfied the following criteria:

- Male students within the ages of 18–25 years
- History of prolonged sitting (≥6 h/day)
- Reduced hamstring extensibility, defined as
  ○ Positive hand–toe test (distance >0 cm)
  ○ Hip flexion angle (HFA) <80° during straight leg raise
- Body Mass Index between 18 and 25 kg/m^2^
- Waist circumference ≤88 cm
- Willingness to comply with the study protocol

#### 2.3.2 Exclusion criteria

Participants were excluded if any of the following were present:

- History of hamstring, spinal, or lower limb injury in the past 3 months
- History of intervertebral disc prolapse, congenital or neurological disorder
- Normal hamstring flexibility (negative hand–toe test)
- Concurrent participation in other research studies

### 2.4 Sample size calculation

Sample size estimation was performed using G*Power software (version 3.1.9.7; Kiel University, Germany). A large effect size (d = 0.8) was assumed based on previous studies reporting substantial effects of massage and stretching interventions on hamstring flexibility (19), type I error rate of 5%, and a statistical power of 80%. In addition, 95% confidence intervals were calculated for key outcome measures and the minimum sample required was calculated as 21 participants per group. Therefore, a total of 42 participants were enrolled in the study.

### 2.5 Randomization and allocation

Eligible participants were randomly allocated into two groups in a 1:1 ratio using a simple randomization technique (lottery method). Sequentially numbered chits (1–42) were prepared and placed in an opaque container. Each participant independently drew a chit without replacement, and allocation to Group A or Group B was based on odd and even numbers, respectively. Allocation concealment was ensured as the group assignment was not known prior to the draw, and the use of an opaque container prevented prediction of allocation. As leg dominance has not been shown to significantly influence muscle stiffness, it was not controlled for, and all outcome measurements were obtained from the right lower limb in all participants (25).

### 2.6 Intervention

Participants in both groups were instructed to continue their usual daily routines, including diet, physical activity, and sleep patterns, throughout the study period.

All participants received initial supervised training, followed by weekly reinforcement sessions to ensure proper technique, duration, and pressure application for both stretching and *Abhyaṅga*. Standardization of pressure was achieved using a sphygmomanometer-based method during training. Adherence to the intervention was monitored through daily follow-up via telephone or direct contact, along with participant-maintained diaries.

#### 2.6.1 Group A: *Nitya-Abhyaṅga* with stretching exercises

Participants in Group A performed standardized hamstring stretching exercises followed by the *Abhyaṅga* protocol using *Tila Taila* (Sesame oil) for 4 weeks. Approximately 10 mL of oil was applied per lower limb. *Abhyaṅga* was performed over the lower limb using uniform longitudinal strokes in the direction of hair growth (*Anuloma Gati*) for a duration of 5 min as described by *Ācārya Dalhana* (900 *Mātrā-kālā*) (26).

Massage pressure was standardized at approximately 2.5 kPa (approximately 18.7 mmHg). Participants were trained to replicate this pressure using a sphygmomanometer cuff inflated to 100 mmHg, applying pressure until the gauge reading increased to approximately 118.7 mmHg. Stroke frequency was trained to be maintained at 20–30 strokes per minute (19).

#### 2.6.2 Group B: stretching exercises alone

Participants in Group B performed only the standardized hamstring stretching exercises for the same 4-week duration.

#### 2.6.3 Stretching exercises protocol

Both groups performed four standardized hamstring stretching exercises: Lying Straight Leg Raise, Lying Gluteal Stretch, Good Morning Hamstring Stretch, and Seated Gluteus Medius Stretch (25).

Each stretch was held for 30 s and repeated three times per session. Stretching was performed once daily.

### 2.7 Outcome measures

All outcome variables were recorded at baseline and again after completion of the 4-week intervention by the primary investigator. Prior to the assessment, participants were instructed to walk at their usual pace for 3 min, followed by 1 min of rest in the supine position. Post-intervention measurements were obtained at least 24 h after the final intervention session to record the long-term effects of massage and stretching exercises and to minimize any acute effects (27, 28).

#### 2.7.1 Hamstring extensibility

##### 2.7.1.1 Hand–toe test

Participants stood barefoot with their heels on the floor and feet positioned hip-width apart. They were asked bend forward as far as possible, while keeping their knees, arms, and fingers fully extended. The vertical distance from the tip of the middle finger to the floor was measured using a measuring tape and recorded to the nearest centimeter (29, 30).

##### 2.7.1.2 Knee extension test

To assess knee extension angle, participants were asked to lie in the supine position with their hip flexed to 90° and ankle relaxed in plantar flexion. They were instructed to actively extend the right knee until hamstring tension was felt, limiting further extension. The angle formed between the long axis of thigh and the leg was measured using a goniometer and defined as the knee extension angle. Movement of the left lower limb was prevented during assessment (31).

##### 2.7.1.3 Hip flexion angle

The hip flexion angle was evaluated using the straight leg raise. Participants were positioned in the supine position with the lower limb fully extended at the knee. They were instructed to actively raise the test limb to the point where discomfort (uncomfortable tension or stretch) was first perceived. The angle between the long axis of the thigh and the torso, using the greater trochanter as the axis of rotation, was measured using a goniometer and recorded as the maximum hip flexion angle (21).

#### 2.7.2 Hamstring stiffness

Hamstring stiffness was assessed using a handheld digital algometer (Biotronix Digital Physiotherapy Algometer with LCD Display; Biotronix, India). Algometers are designed to quantify tissue hardness and pressure pain threshold (32), and their reliability for assessing soft-tissue hardness and stiffness has been previously established (33).

Algometer-derived muscle stiffness was assessed at the hamstring muscle belly with participants in the prone position. The assessment site was standardized at the midpoint between the ischial tuberosity and the medial epicondyle of the tibia. The recorded value represented resistance to transverse compression of the muscle and was used as an index of algometric-derived transverse muscle stiffness, rather than a direct measure of intrinsic viscoelastic muscle stiffness (19, 34, 35).

#### 2.7.3 Stretch tolerance

Stretch tolerance was evaluated using a 100-mm visual analog scale (VAS) at the point of maximum hip flexion angle (36). The scale ranged from “no pain” at the left end to “worst possible pain” at the right end. Participants were asked to draw a vertical line on the scale at the point that best represented the intensity of the pain at maximum HFA. The VAS score was calculated by measuring the distance in millimeters from “no pain” to the participant's mark (19).

### 2.8 Statistical analysis

Data were analyzed using SPSS Statistics software (version 25). Descriptive statistics were reported as mean ± standard deviation (SD) for normally distributed variables, and as median and interquartile range (IQR) for non-normal distributions; ranges were also reported for key outcomes. Normality of the data was tested using the Shapiro–Wilk test to determine the suitability of parametric or non-parametric statistical procedures.

Comparisons between the two groups at baseline and post-intervention were performed using independent-sample *t*-tests for normally distributed variables (e.g., HTT, KET, HFA, stiffness score) and Mann–Whitney *U*-tests for non-normally distributed outcomes (e.g., pain VAS).

A two-way repeated-measures ANOVA was performed with time (pre- and post-intervention) as the within-subject factor and group as the between-subject factor. All hypothesis tests were two-tailed, and a significance level of *p* < 0.05 was considered statistically significant.

## 3 Results

### 3.1 Study participants

A total of 42 healthy male college students were enrolled and randomized equally into Group A (*Nitya-Abhyaṅga* with stretching exercises) and Group B (stretching alone), with 21 participants in each group. All participants were aged 18−25 years and had a normal body mass index (BMI 18−25). All participants demonstrated reduced hamstring extensibility at baseline, as assessed by the hand–toe test and hip flexion angle. Baseline characteristics, such as age, height, weight, waist circumference, and BMI, were comparable between the two groups (*p* > 0.05). Similarly, baseline measures of hamstring extensibility, muscle stiffness, and stretch tolerance did not differ significantly between groups, indicating proper randomization and baseline equivalence. Detailed baseline demographic data are presented in Table 1.

**Table 1:** Baseline demographic data.

Subjects	Group A	Group B	*p*
Age (years)	22.57 ± 1.33	22.24 ± 1.30	0.21
Waist circumference (cm)	83.14 ± 6.68	80.71 ± .05	0.11
Height (cm)	171.57 ± 4.39	170.33 ± 5.78	0.22
Weight (kg)	66.05 ± 5.58	64.57 ± 8.23	0.25
BMI (kg/m^2^)	22.49 ± 1.66	22.18 ± 1.81	0.28
HTT (cm)	17 ± 5.51	15 ± 5.09	0.23
KET (⁰)	41.7 ± 5.3	44.4 ± 3.4	0.06
HFA (⁰)	54.5 ± 6.9	51.1 ± 7.2	0.13
Algometer-derived stiffness (kg/cm^2^)	2.9 ± 0.5	2.8 ± 0.4	0.36
Pain VAS	7 (7–8)	8 (7–8)	0.37

Values are either in mean ± SD or median (IQR).

**Figure 2:**
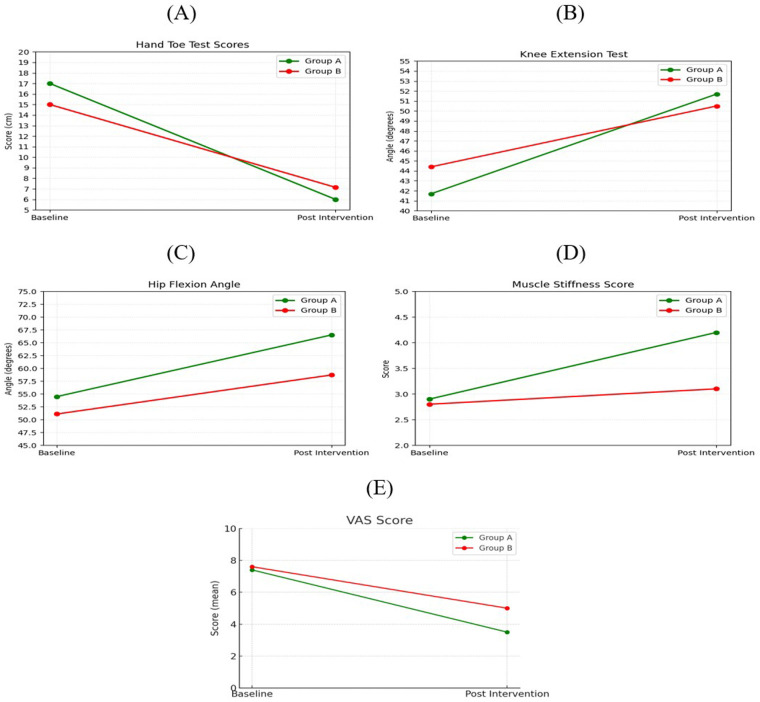
Line graph representation of pre- and post-intervention scores. **(A)** Hand–toe test, **(B)** knee extension test, **(C)** hip flexion angle, **(D)** muscle stiffness, and **(E)** pain VAS score.

All participants completed the study, and no withdrawals were recorded during the intervention period. No adverse event or intervention-related side effects were reported. Compliance was confirmed to be 83.3% ± 3.3% based on participant diaries, indicating good adherence to the protocols. Pre- and post-intervention scores are reported in Table 2 and represented graphically in Figure 2.

**Table 2:** Outcome measures at baseline and post-intervention.

Outcomes	Time	Group A	Group B	*F* (time × group)	*p*-value	Partial *η*^2^
		(*n* = 21)	(*n* = 21)			
HTT (cm)	Baseline	17 ± 5.51	15 ± 5.09	12.04	0.001	0.231
	Post-intervention	6 ± 3.49	7.14 ± 3.72			
KET (°)	Baseline	41.7 ± 5.3	44.4 ± 3.4	32.42	<0.001	0.448
	Post-intervention	51.7 ± 5.8	50.5 ± 4.6			
HFA (°)	Baseline	54.5 ± 6.9	51.1 ± 7.2	32.54	<0.001	0.449
	Post-intervention	66.5 ± 7.9	58.7 ± 8.2			
Algometer-derived stiffness (kg/cm^2^)	Baseline	2.9 ± 0.5	2.8 ± 0.4	223.97	<0.001	0.848
	Post-intervention	4.2 ± 0.5	3.1 ± 0.4			
Pain VAS	Baseline	7 (7–8)	8 (7–8)	44.05	<0.001	0.524
	Post-intervention	4 (3–4)	5 (5–6)			

Values are either in mean ± SD or median (IQR). *p* < 0.05, significant. Statistical analysis was performed using two-way repeated-measures ANOVA. *F*-values represent the interaction effect (time × group).

### 3.2 Hamstring extensibility

#### 3.2.1 Hand–toe test

At baseline, mean hand–toe test (HTT) scores were 17 ± 5.51 cm in Group A and 15 ± 5.09 cm in Group B. Following intervention, HTT scores improved in both groups, with post-intervention values of 6 ± 3.49 cm in Group A and 7.14 ± 3.72 cm in Group B. The between-group mean difference at post-intervention was −1.14 cm (95% CI: −3.39–1.11).

Two-way repeated-measures ANOVA revealed a significant group × time interaction (F(1,40) = 12.04, *p* = 0.001, *η*^2^ = 0.231), indicating that improvement over time was significantly greater in Group A compared with Group B.

#### 3.2.2 Knee extension test

Baseline knee extension angle was 41.7° ± 5.3° in Group A and 44.4° ± 3.4° in Group B. After the 4-week intervention, knee extension angles increased to 51.7° ± 5.8° in Group A and 50.5° ± 4.6° in Group B. The between-group mean difference at post-intervention was 1.14° (95% CI: −2.13–4.41).

A significant group × time interaction was observed (F(1,40) = 32.42, *p* < 0.001, *η*^2^ = 0.448), indicating significantly greater improvement in Group A.

#### 3.2.3 Hip flexion angle

Baseline HFA was 54.5° ± 6.9° in Group A and 51.1° ± 7.2° in Group B. Following the intervention, Group A demonstrated a higher mean HFA of 66.5° ± 7.9°, compared with 58.7° ± 8.2° in Group B. The between-group mean difference at post-intervention was 7.81° (95% CI: −2.78–12.84).

A significant group × time interaction was observed (F(1,40) = 32.54, *p* < 0.001, *η*^2^ = 0.449), indicating significantly greater improvement in proximal hamstring flexibility in Group A.

### 3.3 Muscle stiffness (using an algometer)

Algometric values at baseline were 2.9 ± 0.5 kg/cm^2^ in Group A and 2.8 ± 0.4 kg/cm^2^ in Group B. Following intervention, Group A demonstrated a higher mean algometric score of 4.2 ± 0.5 kg/cm^2^ compared with 3.1 ± 0.4 kg/cm^2^ in Group B. The between-group mean difference at post-intervention was 1.12 kg/cm^2^ (95% CI: −0.82–1.41).

Two-way repeated-measures ANOVA demonstrated a significant group × time interaction (F(1,40) = 223.97, *p* < 0.001, *η*^2^ = 0.848), indicating a markedly greater reduction in algometer-derived muscle stiffness in Group A.

### 3.4 Stretch tolerance

Baseline pain intensity during maximum hip flexion, assessed using the visual analog scale (Pain VAS), was 7.4 ± 0.8 in Group A and 7.6 ± 0.7 in Group B. After the intervention, pain scores decreased in both groups, with Group A demonstrating a lower mean VAS score of 3.5 ± 1.0 compared with 5.0 ± 0.7 in Group B. The between-group mean difference at post-intervention was −1.57 (95% CI: −2.11–1.03).

A significant group × time interaction was observed (F(1,40) = 44.05, *p* < 0.001, *η*^2^ = 0.524), indicating significantly greater improvement in stretch tolerance in Group A.

## 4 Discussion

The present randomized controlled trial investigated the effect of *Nitya-Abhyaṅga* combined with stretching exercises on hamstring extensibility, muscle stiffness, and stretch tolerance in college students with reduced hamstring flexibility. The principal findings indicated that while both groups demonstrated improvements post-intervention, the combination of *Abhyaṅga* and stretching exercises resulted in significantly greater improvements across all outcome measures compared with stretching alone, as evidenced by significant group × time interaction effects using two-way repeated-measures ANOVA.

All parameters—including hand–toe test, knee extension test, hip flexion angle, algometer-derived muscle stiffness, and pain VAS—showed greater improvement in the *Abhyaṅga* plus stretching exercises group, indicating that the addition of *Abhyaṅga* provides a meaningful additive benefit beyond stretching alone.

### 4.1 Hamstring extensibility

Improvements in human muscle extensibility are reflected by increased end-range joint angles. Hamstring extensibility may be assessed using the hand–toe test, knee extension test, and hip flexion angle (37), all of which were recorded in this study.

In the present study, significant improvements were observed across all these parameters—hand–toe test, knee extension test, and hip flexion angle—with moderate to large interaction effects indicating greater improvement in the *Abhyaṅga* with stretching group compared with stretching alone. These findings suggesting that the addition of *Abhyaṅga* enhances both functional and joint-specific components of flexibility beyond stretching alone.

The improvement in extensibility may be attributed to a combination of increased stretch tolerance and reduced passive resistance of the muscle, as proposed by Marek et al. (38).

Similarly, Ylinen et al. reported that repeated performance of multiple hamstring stretches with 30-s holds achieves meaningful gains in extensibility (39), supporting the effectiveness of the stretching protocol used in the present study.

In addition, Lim et al. indicated that improvements in knee extension angle reflect clinically meaningful increases in hamstring length, likely due to reduction in passive stiffness following static stretching when compared with non-exercising control groups (31).

The additional benefits observed with massage may be explained by its influence on soft-tissue properties and pain modulation. Massage has been shown to improve viscoelastic properties of muscle tissue and reduced pain perception, thereby facilitating greater end-range joint motion (40).

Although post-intervention differences in hand–toe test were modest, this measure assesses functional hamstring extensibility, including contributions from the spine, pelvis, hip, and trunk (30). The significant interaction observed across all parameters indicates that improvements were greater in the group performing both *Abhyaṅga* and stretching compared with stretching alone.

### 4.2 Muscle stiffness (using an algometer)

Muscle stiffness in this study was assessed using a handheld digital algometer. According to Fischer, higher algometer values indicate higher pressure thresholds and better tonicity. A muscle pressure threshold of 3 kg or less is generally considered low, while normal ranges lie in the range of 3−6 kg/cm^2^ (41).

Following intervention, the *Abhyaṅga* plus stretching group demonstrated significantly greater improvement in algometer-derived muscle stiffness compared with the stretching alone group. This suggests that massage may reduce tissue resistance, improve tonicity, and reduce algometric-derived muscle stiffness. Crommert et al. proposed that massage may reduce stiffness by decreasing motor–neuron excitability and/or local reflex inhibition in the massaged limb (42) or that massage may break the stable cross-bridges between the actin and myosin filaments spontaneously form at rest (17). Another mechanism, described by Franklin et al., is that increased local circulation and metabolic exchange may reduce nociceptive inputs following massage (43). Moreover, 5 min of massage may increase intramuscular temperature by 2  °C at 1.5 cm depth due to increased circulation and friction during massage, as demonstrated by Drust et al. (44). Given that muscle shear elastic modulus is temperature-dependent, this rise in temperature could be one of the reasons for reduced stiffness in muscle (42). However, these mechanisms were not directly assessed in the present study and should be interpreted as theoretical explanations for the benefits of massage combined with stretching exercises.

The assessment of muscle stiffness in the present study was done using an algometer. Algometer-derived measures reflect resistance to external compression (tissue hardness) rather than intrinsic viscoelastic stiffness. Therefore, findings should be interpreted as changes in algometer-derived muscle stiffness rather than absolute biomechanical stiffness.

### 4.3 Stretch tolerance

Stretch tolerance, assessed using the visual analog scale during maximum hip flexion, improved significantly in the *Abhyaṅga* plus stretching group compared with stretching alone. The observed improvement in stretch tolerance may be explained by previous studies. Furlan et al. suggested that massage was better than active control for symptomatic relief of pain through the release of endorphins (45). Furthermore, the gate control theory suggests that massaging a particular area stimulates large-diameter nerve fibers. These fibers have an inhibitory response in the dorsal horn when subjected to non-painful inputs. These interneurons “close the gate” on the smaller pain fibers, thereby reducing the transmission of pain signals (46).

### 4.4 Ayurvedic interpretation

From an Ayurvedic perspective, reduced muscle extensibility and increased stiffness may be correlated with *Saṅkocha* (contracture) and *Stambha* (rigidity) due to localized vitiation of *Vāta Doṣa* (22). *Abhyaṅga*, described as a part of *Dinacharya*, is indicated for maintaining musculoskeletal health and preventing *Vāta-*related disorders (23).

The observed improvements in hip flexion angle, algometer-derived muscle stiffness, and stretch tolerance in the present study may be interpreted in terms of *mardavata* (softness), *dr̥ḍhatā* (tonic stability), and reduced *stabdata* (stiffness), thereby alleviating *Saṅkocha* and *stambha* and improving pliability and functional mobility (24).

Ācārya Dalhana further notes that 5 min of massage nourishes all the *dhatus*, which may be correlated with improved circulation and metabolic exchange. This in turn reduces nociceptive inputs from the muscle, thereby enhancing muscle extensibility through restoration of functional mobility (43).

The practice of *Abhyaṅga* in *anuloma gati* (massage strokes in the direction of hair follicles) is described to facilitate the proper flow of *Vāta* and removal of *srotorodha* (channel obstruction). This may be correlated with the breaking of resting stable actin-myosin cross-bridges during soft-tissue manipulation such as massage (17). *Kleśa-Vyāyāma-Samsaha* (improved tolerance to discomfort and fatigue), explained by Ācārya Charaka, may be linked to the analgesic effect of massage, which can be explained as symptomatic relief of pain through the release of endorphins (45) or the gate control theory of pain (46). Thus, the findings of this study may be viewed as an integrative outcome of both Ayurvedic principles and modern physiological mechanisms, supporting the additive role of *Abhyaṅga* when combined with stretching.

## 5 Conclusion

In the present era, sedentary lifestyles and prolonged sitting have become increasingly prevalent among university students, predisposing them to musculoskeletal injuries such as hamstring stiffness, reduced flexibility, and postural instability. Optimal hamstring extensibility and appropriate muscle tone are essential for maintaining functional movement, postural alignment, and prevention of lower back strain.

This study evaluated the effect of *Nitya-Abhyaṅga* with *Tila Taila* in combination with standard stretching exercises on hamstring extensibility, stiffness, and stretch tolerance among healthy male college students. The findings demonstrated that participants who performed *Abhyaṅga* in addition to stretching showed a significant reduction in algometer-derived stiffness and improvement in hamstring extensibility and stretch tolerance compared with the stretching-only group, as evidenced by significant group × time interaction effects.

The additional benefits observed in the *Abhyaṅga* along with stretching group may be attributed to its influence on tissue pliability by imparting *Mārdavata* (softness), *Dr̥ḍhatā* (tonic stability), and *Bala* (strength), while reducing *Stambha* (stiffness) and *Saṅkocha* (contracture). These effects are explained through the *Vāta-hara*, *Snigdha*, *and Kleśa-Vyāyāma-Samsaha* properties of *Abhyaṅga*.

Given its simplicity and low cost, *Nitya-Abhyaṅga* combined with stretching exercises may be recommended as a supportive *Dinācaryā* practice for maintaining musculoskeletal health, improving postural stability, and potentially reducing the risk of lower back pain and related disorders among sedentary college students.

## 6 Limitation and future prospects

This study included only healthy male participants aged 18–25 years from a single institution, which limits the generalizability of the findings to females, other age groups, and clinical populations. While the use of a relatively homogeneous sample may have reduced variability and improved internal validity, future studies should include more diverse demographic and clinical populations to validate and extend these findings. The study was conducted in an open-label manner without participant or assessor blinding. This may introduce both performance and detection bias, particularly for subjective outcomes such as stretch tolerance (VAS). Although standardized protocols and objective outcome measures were used to minimize bias, the potential influence of expectation bias cannot be completely ruled out. The absence of a true no-treatment control group and an *Abhyaṅga*-only intervention group limits the ability to isolate the independent effects of *Abhyaṅga* from those of stretching. The assumption of a large effect size may have overestimated the true treatment effect, potentially limiting the power to detect smaller differences. Inclusion of these comparator groups in the future studies would allow a clearer delineation of the specific contribution of *Abhyaṅga* in extensibility, stiffness, and stretch tolerance. Although algometry has previously been validated against electromyography (47), we acknowledge that it has not yet been directly validated against gold-standard techniques such as ultrasound elastography or infrared thermography. Algometer primarily measures resistance to external compression and pressure sensitivity, reflecting tissue hardness rather than intrinsic viscoelastic muscle stiffness. Therefore, the stiffness values obtained in this study should be interpreted as indirect indices of transverse muscle stiffness rather than direct measures of muscle viscoelastic properties. Incorporation of these advanced biomechanical and imaging modalities in future studies may provide more objective assessments of changes in muscle elasticity, neural activation, and tissue temperature. Although regular follow-up and periodic reinforcement were conducted, the intervention was largely self-administered, and variations in technique and pressure application cannot be completely ruled out.

## Data Availability

The original contributions presented in the study are included in the article/Supplementary Material; further inquiries can be directed to the corresponding author.

## References

[1] Woodley SJ Mercer SR. Hamstring muscles: architecture and innervation. Cells Tissues Organs. (2005) 179(3):125–41. 10.1159/000085004 15947463

[2] Rodgers CD Raja A. Anatomy, bony pelvis and lower limb, hamstring muscle. In: StatPearls. Treasure Island (FL): StatPearls Publishing (2025). Available online at: http://www.ncbi.nlm.nih.gov/books/NBK546688/ (Accessed October 17, 2025). 31536294

[3] Bradley PS Portas MD. The relationship between preseason range of motion and muscle strain injury in elite soccer players. J Strength Cond Res. (2007) 21(4):1155–9. 10.1519/R-20416.1 18076233

[4] Kuszewski M Gnat R Sobota G Myśliwiec A. Influence of passive stiffness of hamstrings on postural stability. J Hum Kinet. (2015) 45:49–57. 10.1515/hukin-2015-0006 25964809 PMC4415843

[5] Park JH Moon JH Kim HJ Kong MH Oh YH. Sedentary lifestyle: overview of updated evidence of potential health risks. Korean J Fam Med. (2020) 41(6):365–73. 10.4082/kjfm.20.0165 33242381 PMC7700832

[6] Fatima G Qamar M Ul Hassan J Basharat A. Extended sitting can cause hamstring tightness. Saudi J Sports Med. (2017) 17(2):110. 10.4103/sjsm.sjsm_5_17

[7] Naqvi R Arshad N Imran M Aftab AA Batool S. Prevalence of hamstring tightness among university students in Lahore, Pakistan. Rawal Med J. (2019) 44(4):853.

[8] Cross KM Gurka KK Conaway M Ingersoll CD. Hamstring strain incidence between genders and sports in NCAA athletics. Athl Train Sports Health Care. (2010) 2(3):124–30. 10.3928/19425864-20100428-06

[9] Yadav R Basista R. Effect of prolonged sitting on hamstring muscle flexibility and lumbar lordosis in collegiate student. Int J Health Sci Res. (2020) 10(9):280–9.

[10] Wisdom KM Delp SL Kuhl E. Use it or lose it: multiscale skeletal muscle adaptation to mechanical stimuli. Biomech Model Mechanobiol. (2015) 14(2):195–215. 10.1007/s10237-014-0607-3 25199941 PMC4352121

[11] Goto K Okuyama R Honda M Uchida H Akema T Ohira Y. Profiles of connectin (titin) in atrophied soleus muscle induced by unloading of rats. J Appl Physiol. (2003) 94(3):897–902. 10.1152/japplphysiol.00408.2002 12391127

[12] Gajdosik RL. Passive extensibility of skeletal muscle: review of the literature with clinical implications. Clin Biomech. (2001) 16(2):87–101. 10.1016/S0268-0033(00)00061-9 11222927

[13] Smith LR Lee KS Ward SR Chambers HG Lieber RL. Hamstring contractures in children with spastic cerebral palsy result from a stiffer extracellular matrix and increased *in vivo* sarcomere length. J Physiol. (2011) 589(10):2625–39. 10.1113/jphysiol.2010.203364 21486759 PMC3115830

[14] Kjær M. Role of extracellular matrix in adaptation of tendon and skeletal muscle to mechanical loading. Physiol Rev. (2004) 84(2):649–98. 10.1152/physrev.00031.2003 15044685

[15] Boukabache A Preece SJ Brookes N. Prolonged sitting and physical inactivity are associated with limited hip extension: a cross-sectional study. Musculoskelet Sci Pract. (2021) 51:102282. 10.1016/j.msksp.2020.102282 33188982

[16] Hill DK. Tension due to interaction between the sliding filaments in resting striated muscle. The effect of stimulation. J Physiol (Lond). (1968) 199(3):637–84. 10.1113/jphysiol.1968.sp008672 5710425 PMC1365364

[17] Proske U Morgan DL. Do cross-bridges contribute to the tension during stretch of passive muscle? J Muscle Res Cell Motil. (1999) 20(5–6):433–42. 10.1023/a:1005573625675 10555062

[18] Herbert RD de Noronha M Kamper SJ. Stretching to prevent or reduce muscle soreness after exercise. Cochrane Database Syst Rev. (2011) (4):CD004577. 10.1002/14651858.CD004577 21735398

[19] Akazawa N Okawa N Kishi M Nakatani K Nishikawa K Tokumura D. Effects of long-term self-massage at the musculotendinous junction on hamstring extensibility, stiffness, stretch tolerance, and structural indices: a randomized controlled trial. Phys Ther Sport. (2016) 21:38–45. 10.1016/j.ptsp.2016.01.003 27428533

[20] Hodgson DD Quigley PJ Whitten JH Reid JC Behm DG. Impact of 10-minute interval roller massage on performance and active range of motion. J Strength Cond Res. (2019) 33(6):1512–23. 10.1519/JSC.0000000000002271 29189581

[21] Huang SY Di Santo M Wadden KP Cappa DF Alkanani T Behm DG. Short-duration massage at the hamstrings musculotendinous junction induces greater range of motion. J Strength Cond Res. (2010) 24(7):1917–24. 10.1519/JSC.0b013e3181e06e0c 20543728

[22] Vaidya Jadavji Trikamji Acharya. Charaka Samhita by Agnivesha Chikitsa Sthana Chapter 28 Shloka no:20. 2010th ed. Varanasi: Chaukhamba orientalia (2010) 617.

[23] Bhisagacharya Harisastri Paradakara Vaidyya. Ashtanga Hridaya by Vagbhata, Sutrasthanam Chapter 2 Shloka no 9. 9th edn. Bombay: Pandurang Jawaji (2005).

[24] Srikantha Murthy KR. Ashtanga Sangraha of Vagbhata, Sutrasthanam Chapter 3 Shloka 31. Varanasi: Chaukhamba orientalia (1999).

[25] Marshall PWM Cashman A Cheema BS. A randomized controlled trial for the effect of passive stretching on measures of hamstring extensibility, passive stiffness, strength, and stretch tolerance. J Sci Med Sport. (2011) 14(6):535–40. 10.1016/j.jsams.2011.05.003 21636321

[26] Acharya Yadavji Trikamji. Suśruta Samhita of Suśruta with Nibandha Commentary of Dalhana, Chikitsa Sthana Chapter 24 Shloka 30. Varanasi: Chaukhamba Sanskrit Series Office (1938) 109–14.

[27] Law RYW Harvey LA Nicholas MK Tonkin L De Sousa M Finniss DG. Stretch exercises increase tolerance to stretch in patients with chronic musculoskeletal pain: a randomized controlled trial. Phys Ther. (2009) 89(10):1016–26. 10.2522/ptj.20090056 19696119

[28] Folpp H Deall S Harvey LA Gwinn T. Can apparent increases in muscle extensibility with regular stretch be explained by changes in tolerance to stretch? Aust J Physiother. (2006) 52(1):45–50. 10.1016/S0004-9514(06)70061-7 16515422

[29] Ayala F Sainz de Baranda P De Ste Croix M Santonja F. Reproducibility and criterion-related validity of the sit and reach test and toe touch test for estimating hamstring flexibility in recreationally active young adults. Phys Ther Sport. (2012) 13(4):219–26. 10.1016/j.ptsp.2011.11.001 23068896

[30] Merritt JL McLean TJ Erickson RP Offord KP. Measurement of trunk flexibility in normal subjects: reproducibility of three clinical methods. Mayo Clin Proc. (1986) 61(3):192–7. 10.1016/S0025-6196(12)61848-5 3945120

[31] Lim KI Nam HC Jung KS. Effects on hamstring muscle extensibility, muscle activity, and balance of different stretching techniques. J Phys Ther Sci. (2014) 26(2):209–13. 10.1589/jpts.26.209 24648633 PMC3944290

[32] Somphithak S Chatchawan U Pimdee A Sucharit W. Reliability and responsiveness of a tissue hardness meter and algometer for measuring tissue hardness and pressure pain threshold in upper trapezius myofascial trigger points. PeerJ. (2025) 13:e19580. 10.7717/peerj.19580 40511384 PMC12161125

[33] Pelfort X Torres-Claramunt R Sánchez-Soler JF Hinarejos P Leal-Blanquet J Valverde D. Pressure algometry is a useful tool to quantify pain in the medial part of the knee: an intra- and inter-reliability study in healthy subjects. Orthop Traumatol Surg Res. (2015) 101(5):559–63. 10.1016/j.otsr.2015.03.016 26025162

[34] Murayama M Nosaka K Yoneda T Minamitani K. Changes in hardness of the human elbow flexor muscles after eccentric exercise. Eur J Appl Physiol. (2000) 82(5):361–7. 10.1007/s004210000242 10985588

[35] Arokoski JPA Surakka J Ojala T Kolari P Jurvelin JS. Feasibility of the use of a novel soft tissue stiffness meter. Physiol Meas. (2005) 26(3):215. 10.1088/0967-3334/26/3/007 15798297

[36] Bijur PE Silver W Gallagher EJ. Reliability of the visual analog scale for measurement of acute pain. Acad Emerg Med. (2001) 8(12):1153–7. 10.1111/j.1553-2712.2001.tb01132.x 11733293

[37] Weppler CH Magnusson SP. Increasing muscle extensibility: a matter of increasing length or modifying sensation? Phys Ther. (2010) 90(3):438–49. 10.2522/ptj.20090012 20075147

[38] Marek SM Cramer JT Fincher AL Massey LL Dangelmaier SM Purkayastha S. Acute effects of static and proprioceptive neuromuscular facilitation stretching on muscle strength and power output. J Athl Train. (2005) 40(2):94–103. 15970955 PMC1150232

[39] Ylinen JJ Kautiainen HJ Häkkinen AH. Comparison of active, manual, and instrumental straight leg raise in measuring hamstring extensibility. J Strength Cond Res. (2010) 24(4):972. 10.1519/JSC.0b013e3181d0a55f 20300030

[40] Ateş R Yaşar P Başkurt F Başkurt Z Ercan S. A comparison of the acute effects of percussion massage therapy and static stretching on hamstring elasticity. Ethiop J Health Sci. (2023) 33(4):695–702. 10.4314/ejhs.v33i4.16 38784209 PMC11111184

[41] Fischer AA. Pressure algometry over normal muscles. Standard values, validity and reproducibility of pressure threshold. Pain. (1987) 30(1):115–26. 10.1016/0304-3959(87)90089-3 3614975

[42] Eriksson Crommert M Lacourpaille L Heales LJ Tucker K Hug F. Massage induces an immediate, albeit short-term, reduction in muscle stiffness. Scand J Med Sci Sports. (2015) 25(5):e490–6. 10.1111/sms.12341 25487283

[43] Franklin NC Ali MM Robinson AT Norkeviciute E Phillips SA. Massage therapy restores peripheral vascular function following exertion. Arch Phys Med Rehabil. (2014) 95(6):1127–34. 10.1016/j.apmr.2014.02.007 24583315 PMC4037335

[44] Drust B Atkinson G Gregson W French D Binningsley D. The effects of massage on intra muscular temperature in the vastus lateralis in humans. Int J Sports Med. (2003) 24(6):395–9. 10.1055/s-2003-41182 12905085

[45] Furlan AD Giraldo M Baskwill A Irvin E Imamura M. Massage for low-back pain. Cochrane Database Syst Rev. (2015) 2015(9):CD001929. 10.1002/14651858.CD001929.pub3 26329399 PMC8734598

[46] Melzack R Wall PD. Pain mechanisms: a new theory. Science. (1965) 150(3699):971–9. 10.1126/science.150.3699.971 5320816

[47] Morozumi K Fujiwara T Karasuno H Morishita K Castel C Palermo FX. A new tissue hardness meter and algometer; a new meter incorporating the functions of a tissue hardness meter and an algometer. J Phys Ther Sci. (2010) 22(3):239–45. 10.1589/jpts.22.239

